# Health Tract, No. 53

**Published:** 1865

**Authors:** 


### HEALTH TRACT, No. 53.

### FIFTEEN FOLLIES.

1. To think that the more a man eats the fatter and stronger he will become.

2. To believe that the more hours children study at school the faster they learn.

3. To conclude that if exercise is good for the health, the more violent and exhausting it is, the more good is done.

4. To imagine that every hour taken from sleep is an hour gained.

5. To act on the presumption that the smallest room in the house is large enough to sleep in.

6. To argue that whatever remedy causes one to feel immediately better, is "good for" the system without regard to more ulterior effects. The "soothing syrup," for example, does stop the cough of children, and does arrest diarrhea, only to cause, a little later, alarming convulsions, or the more fatal inflammation of the brain, or water on the brain ; at least, always protracts the disease.

7. To commit an act which is felt in itself to be prejudicial, hoping that some how or other it may be done in your case with impunity.

8. To advise another to take a remedy which you have not tried on yourself, or without making special inquiry whether all the conditions are alike.

9. To eat without an appetite, or continue to eat after it has been satiated, merely to gratify the taste.

10. To eat a hearty supper for the pleasure experienced during the brief time it is passing down the throat, at the expense of a whole night of disturbed sleep, and a weary waking in the morning.

11. To remove a portion of the clothing immediately after exercise, when the most stupid drayman in New-York knows that if he does not put a cover on his horse the moment he ceases work in winter, he will lose him in a few days by pneumonia.

12. To contend that because the dirtiest children in the street, or on the highway, are hearty and healthy, that, therefore, it is healthy to be dirty; forgetting that continuous daily exposure to the pure out-door air, in joyous, unrestrained activities, is such a powerful agency for health that those who live thus are well, in spite of rags and filth.

13. To presume to repeat, later in life, without injury, the indiscretions, exposures, and intemperances which in the flush of youth were practiced with impunity.

14. To believe that warm air is necessarily impure, or that pure, cool air is necessarily more healthy than the confined air of a close and crowded vehicle; the latter, at most, can only cause fainting or nausea; while entering a conveyance after walking briskly, lowering a window, thus while still, exposed to a draught, will give a cold infallibly, or an attack of pleurisy or pneumonia, which will cause weeks and months of suffering, if not actual death within four days.

15. To "Remember the Sabbath-day" by working harder and later on Saturday than on any other day in the week, with a view to sleeping late next morning, and staying at home all day to rest, conscience being quieted by the plea of not "feeling very well."

